# High-Responsivity UV–Blue Photodetector Based on Nanostructured CdS and Prepared by Solution Processing

**DOI:** 10.3390/nano15161212

**Published:** 2025-08-08

**Authors:** Jian-Ru Lai, Fang-Hsing Wang, Han-Wen Liu, Tsung-Kuei Kang

**Affiliations:** 1 Department of Electrical Engineering, National Chung Hsing University, Taichung 40227, Taiwan; 2 Graduate Institute of Optoelectronic Engineering, National Chung Hsing University, Taichung 40227, Taiwan; 3 Department of Electronic Engineering, Feng-Chia University, Taichung 40724, Taiwan; kangtk@o365.fcu.edu.tw

**Keywords:** CdS thin films, nanostructure, chemical bath deposition, UV–blue photodetectors, CuSCN, p–n heterojunction, high responsivity

## Abstract

Ultraviolet (UV) and blue-light photodetectors are vital in environmental monitoring, medical and biomedical applications, optical communications, and security and anti-counterfeiting technologies. However, conventional silicon-based devices suffer from limited sensitivity to short-wavelength light due to their narrow indirect bandgap. In this study, we investigate the influence of precursor concentration on the structural, optical, and photoresponse characteristics of nanostructured CdS thin films synthesized via chemical bath deposition. Among the CdS samples prepared at different precursor concentrations, the best photoresponsivity of 21.1 mA/W was obtained at 2 M concentration. Subsequently, a p–n heterojunction photodetector was fabricated by integrating a spin-coated CuSCN layer with the optimized CdS nanostructure. The resulting device exhibited pronounced rectifying behavior with a rectification ratio of ~750 and an ideality factor of 1.39. Under illumination and a 5 V bias, the photodetector achieved an exceptional responsivity exceeding 10^4^ A/W in the UV region—over six orders of magnitude higher than that of CdS-based metal–semiconductor–metal devices. This remarkable enhancement is attributed to the improved light absorption, efficient charge separation, and enhanced hole transport enabled by CuSCN incorporation and heterojunction formation. These findings present a cost-effective, solution-processed approach to fabricating high-responsivity nanostructured photodetectors, promising for future applications in smart healthcare, environmental surveillance, and consumer electronics.

## 1. Introduction

Photodetectors are essential components widely used in modern technologies such as smartphones, tablets, security systems, biomedical monitoring devices, industrial automation, and environmental sensing systems. Currently, most commercial photodetectors are fabricated using single-crystalline silicon, which enables broadband light detection on a spectrum from ultraviolet (UV) to near-infrared (NIR) (190–1100 nm). Silicon-based devices benefit from mature fabrication technologies and seamless integration with CMOS circuitry. However, due to silicon’s narrow and indirect bandgap (~1.1 eV), its internal gain and sensitivity to short-wavelength light are limited.

Excessive exposure to ultraviolet radiation has been associated with various health risks, including cataracts, skin cancer, and accelerated aging [1,2]. Similarly, blue light (400–500 nm), commonly emitted by high-color-temperature sources such as smartphones, computer monitors, and LED lighting, has been shown to negatively impact eye health. Blue light can induce the formation of reactive oxygen species in retinal cells, leading to the degeneration of retinal pigment epithelial (RPE) cells, potentially causing irreversible vision loss [3,4]. Consequently, the development of high-performance short-wavelength photodetectors is becoming increasingly important in today’s light-intensive environment.

Numerous studies have focused on UV photodetectors [5,6,7,8,9,10]. For instance, A. Hosseini et al. reported a flexible UV photodetector fabricated using silver-doped ZnO nanostructures via a drop-casting method, achieving a responsivity of 17.3 mA/W at 5 V bias under 57 µW illumination [6]. Shashi Pandey et al. demonstrated a high-performance UV photodetector based on a ZnO/Ga_2_O_3_ heterojunction, prepared through spin-coating and hydrothermal processing, with a maximum responsivity of 38 A/W at ~200 nm under a 10 V bias [5].

In contrast, blue-light photodetectors are less commonly reported, and many utilize organic materials such as perovskites or require expensive fabrication techniques like molecular beam epitaxy (MBE) [11,12,13,14,15,16]. For example, Wu, Zhi-Cheng et al. reported a lead-free perovskite MA_3_Bi_2_Br_9_-based blue-light photodetector with a responsivity of 0.102 mA/W under 470 nm illumination at 15 V bias [12]. Chen, Liang et al. demonstrated a blue-light photodetector based on a Ag nanowire-decorated InGaN nanorod/PEDOT/PSS heterojunction, achieving a responsivity of 2.9 A/W under 420 nm illumination at 1 V bias [15].

In this study, we present a low-cost, solution-processed photodetector capable of detecting short-wavelength light, aiming to address the limitations of conventional silicon- and organic-based short-wavelength detectors, which are often costly, fabrication-intensive, and limited in performance. Specifically, we present a high-responsivity photodetector based on a p-n heterojunction structure. Compared to a standard metal–semiconductor–metal (MSM) device, the heterojunction architecture demonstrates a responsivity enhancement by a factor of 10^6^. Our experimental results demonstrate that this novel and low-cost photodetector significantly outperforms traditional photodetectors in terms of both responsivity and detectivity. 

## 2. Materials and Methods

### 2.1. Material Synthesis and Device Fabrication

To synthesize CdS nanostructured thin films, a two-step approach combining sol–gel and chemical bath deposition (CBD) techniques was employed. This process involved sequential deposition of a CdS buffer layer followed by the primary nanostructured CdS layer. Glass substrates (AT35EX) and ITO-coated glass substrates (2 cm × 5 cm), both supplied by Ruilong Optoelectronics (Miaoli, Taiwan), were used. Prior to deposition, the substrates were ultrasonically cleaned in acetone, isopropanol, and deionized water to remove surface contaminants such as grease, dust, and organic residues.

The CdS thin film was fabricated through a two-step deposition process. Initially, the buffer layer was deposited by the sol–gel spin-coating method, followed by CBD growth of the nanostructured CdS layer. The detailed procedures and parameters were as follows:

Cadmium acetate dihydrate (1.6 g, 98%, Acros Organics, Geel, Belgium) and thiourea (0.46 g, 99.9%, Ultimate Materials Tech., New Taipei City, Taiwan) were separately dissolved in 10 mL of 2-methoxyethanol (99%, Alfa Aesar, Heysham, UK). The solutions were stirred separately at room temperature for 2 h at 300 rpm until fully dissolved, then combined to form a 0.3 M precursor solution. Subsequently, ethanolamine (0.5 mL, 99.4%, Ultimate Materials Tech.) was added, and the mixture was aged for 4 h at room temperature. During the aging process, the solution gradually changed from colorless and transparent to pale yellow, forming a stable CdS sol.

The CdS buffer layer was deposited via a two-step spin-coating procedure at 500 rpm for 10 s and 3000 rpm for 20 s. The coated substrates were then heated on a hot plate at 200 °C for 10 min to remove residual solvents. This spin-coating and annealing process was repeated three times to form a three-layer film. Subsequent annealing was performed in a tube furnace under vacuum at 400 °C for 1 h.

The nanostructured CdS layer was subsequently deposited by CBD using an ammonia-based aqueous solution. Equimolar cadmium sulfate (99+%, Thermo Scientific, Waltham, MA, USA) and thiourea served as precursors [17]. The chemical bath was maintained at 80 °C for 3 h. To investigate the influence of precursor concentration on film properties, samples were prepared with different precursor molarities (1, 1.5, and 2 M). After deposition, the samples were annealed in a nitrogen atmosphere at 300 °C for 1 h, followed by natural cooling to room temperature.

The copper(I) thiocyanate (CuSCN, 99%, Macklin, Shanghai, China) thin film was prepared by dissolving CuSCN powder (30 mg/mL) in dimethyl sulfoxide (DMSO, 99.8%, Emperor Chemical, Taipei, Taiwan) [18,19]. The solution was stirred at 600 rpm and heated to 120 °C for 24 h to ensure complete dissolution. The CuSCN layer was then deposited using a two-step spin-coating process, followed by heating at 100 °C for 10 min to remove residual solvents. This coating process was repeated 20 times to achieve the desired film thickness. Patterning of the CuSCN layer was accomplished via photolithography and wet etching. Finally, gold/titanium (Au/Ti) electrodes were deposited using e-beam evaporation through a shadow mask.

### 2.2. Material and Device Performance Characterization

Structural properties of the thin films were characterized using X-ray diffraction (XRD, Bruker D8 Discover, Ettlingen, Germany). Surface morphology of the nanostructured CdS film was examined using a field-emission scanning electron microscope (FESEM, Hitachi S-4800, Tokyo, Japan). Optical transmittance was measured using a UV–Vis spectrophotometer (Thermo Fisher Scientific Evolution 220, Waltham, MA, USA). The current–voltage characteristics were measured using a source meter (Keithley 2450, Solon, OH, USA). Photocurrent measurements were conducted using a 150 W xenon lamp light source (Beijing Saifan Optoelectronics, 7ILX150A-UVC, Beijing, China) coupled with a monochromator (Beijing Saifan Optoelectronics, 7IMU1021, Beijing, China).

## 3. Results and Discussion

### 3.1. Nanostructured CdS Thin Films

#### 3.1.1. Structural and Optical Properties

**Figure 1:**
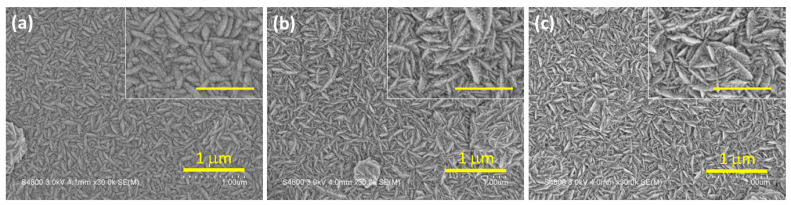
Plan-view FESEM images of nanostructured CdS thin films prepared at various concentrations: (**a**) 1 M, (**b**) 1.5 M, and (**c**) 2 M. The inset shows the magnified micrograph, and the scale bar represents a length of 500 nm.

Figure 1 shows the surface morphology of nanostructured CdS thin films deposited via CBD with varying precursor concentrations. The corresponding high-magnification images are displayed as insets. At a precursor concentration of 1 M, the film exhibits worm-like nanostructures distributed across the surface, as shown in Figure 1a. As the precursor concentration increases to 1.5 M and 2 M, the surface morphology undergoes a significant transformation, evolving into densely packed, pine needle-like nanostructures, as shown in Figure 1b and Figure 1c, respectively. This morphological evolution can be attributed to the enhanced nucleation and growth rates at higher precursor concentrations. At a lower concentration (1 M), the limited availability of Cd^2+^ and S^2−^ ions results in the formation of discrete, relatively isotropic nanostructures. In contrast, at higher concentrations, increased supersaturation drives anisotropic crystal growth, promoting the development of thinner, needle-like features. This behavior differs from previously reported CBD-grown CdS thin films, where lower precursor concentration led to round-shaped grains or uniform and compact morphology [20,21]. The pine needle-like surface texture observed here increases the effective surface area and facilitates enhanced light scattering and absorption, which is beneficial for photodetector applications.

**Figure 2:**
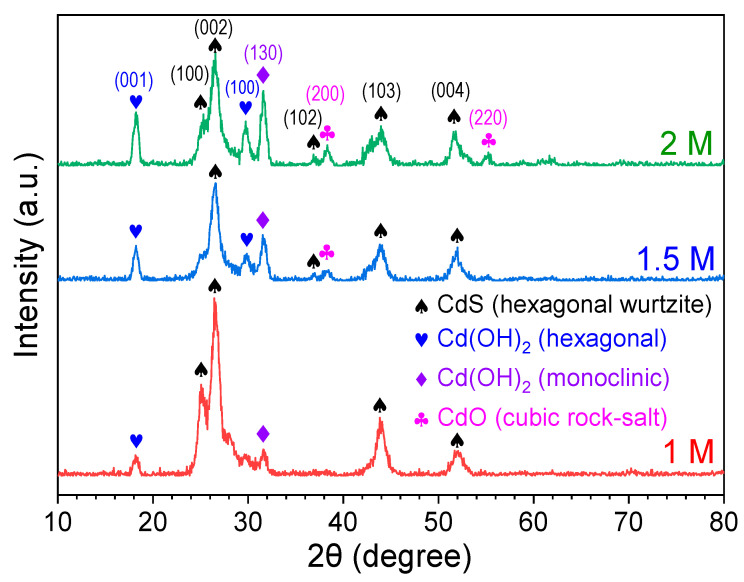
XRD patterns of CdS thin films deposited at various concentrations.

Figure 2 displays the X-ray diffraction (XRD) patterns of CdS thin films prepared with different precursor concentrations. All films exhibit polycrystalline characteristics with a complex microstructural composition. Prominent diffraction peaks are observed at 2θ = 25.32°, 26.56°, 36.99°, 44.04°, and 51.79°, corresponding to the (100), (002), (102), (103), and (004) planes of the hexagonal wurtzite phase of CdS, respectively (JCPDS No. 061-0314) [22]. Additional peaks at 2θ = 18.21° and 29.76°, indexed to the (001) and (100) planes, respectively, are attributable to hexagonal cadmium hydroxide (Cd(OH)_2_), as referenced by JCPDS No. 31-0228 [23]. The diffraction peak at 2θ = 31.72°, corresponding to the (130) plane, is associated with monoclinic Cd(OH)_2_ (JCPDS No. 84-1767) [24]. Furthermore, peaks at 2θ = 38.32° and 55.28°, associated with the (200) and (220) planes, respectively, are assigned to the cubic rock-salt phase of cadmium oxide (CdO) (JCPDS No. 05-0640) [24].

A comparative analysis of the XRD patterns reveals that the intensity of Cd(OH)_2_-related peaks increases slightly with higher precursor concentrations, alongside the emergence of CdO peaks. This structural evolution is attributed to the enhanced formation of Cd(OH)_2_ at elevated precursor concentration and higher pH, which subsequently dehydrates to form CdO. These findings are consistent with previous reports by M. Shkir and T. Alshahrani [25], as well as Ruiz-Ortega et al. [26], which indicate that CdS films deposited at lower concentrations primarily exhibit a hexagonal wurtzite structure.

To further elucidate the crystallographic characteristics of CdS, the full width at half maximum (FWHM, *β*) of the (002) diffraction peak was utilized to calculate the crystallite size (*D*) using the Scherrer equation (Equation (1)) [27]. Dislocation density (*δ*) and microstrain (*ε*) were subsequently estimated using the following relationships (Equations (2) and (3)) [26,27], where *λ* = 1.54056 Å, *θ* is the Bragg angle, and *β* is the FWHM.

(1) $$D=\frac{0.9\lambda }{\beta \cos\theta }$$

(2) $$\delta =\frac{1}{D^{2}}$$

(3) $$\varepsilon =\frac{\beta }{4\tan\theta }$$

The detailed crystallographic parameters of the CdS thin films are summarized in Table 1. As the precursor concentration increases, a systematic shift in the (002) diffraction peak toward lower angles is observed. At the same time, the FWHM of the (002) peak decreases, indicating an increase in crystallite size at higher concentrations. Additionally, both dislocation density and microstrain exhibit a slight reduction with increasing concentration. These results suggest that higher chemical bath concentrations facilitate the growth of larger, better-ordered crystallites, thereby enhancing the overall crystalline quality of the hexagonal CdS films.

**Table 1:** XRD parameters of CdS thin films deposited at various concentrations.

Sample	2θ (°)	FWHM (°)	D (nm)	δ (×10^−2^ nm^−2^)	ε (×10^−3^)
1 M	26.62	1.092	7.48	1.79	5.18
1.5 M	26.62	1.017	8.03	1.55	4.82
2 M	26.56	0.9709	8.41	1.41	4.89

**Figure 3:**
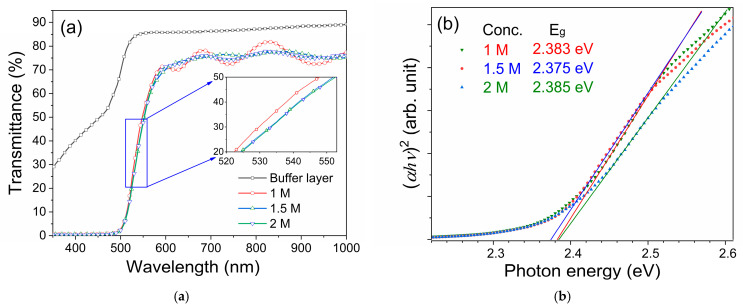
(**a**) Transmittance spectra and (**b**) Tauc plot of CdS thin films deposited under various precursor concentrations.

Figure 3a presents the transmittance spectra of CdS thin films deposited with different precursor concentrations. All samples exhibit a sharp absorption edge in the wavelength range of 510–550 nm. In the visible region beyond 550 nm (550–800 nm), the films maintain a high average transmittance of approximately 70% to 80%, indicating good transparency. In contrast, for wavelengths below 500 nm, the transmittance rapidly drops to less than 1%, indicating strong optical absorption in the short-wavelength spectral regions.

The optical bandgap (*E_g_*) of the CdS films was estimated using the Tauc plot method, which is based on the following equation [28]:

(4) (α*hυ*)*^n^* = *C*(*hυ* − *E_g_*)

where *α* is the absorption coefficient, *hν* is the photon energy (the product of Planck’s constant and frequency), *C* is a material-dependent constant, and *n* = 2 for direct bandgap semiconductors such as CdS.

From the three samples obtained at each precursor concentration, the sample with an optical bandgap value closest to the average was selected for analysis, as presented in Figure 3b. The optical bandgaps of the CBD-grown CdS films show a slight deviation from the bulk CdS value of 2.4 eV. Notably, the band gap of the film deposited using the 1.5 M precursor solution is slightly lower than that of the other films. This slight decrease in the band gap value (about 0.4%) is within the reasonable range for manual solution processing and/or may be attributed to the formation of sulfur vacancies and surface states, which can introduce localized energy levels near the conduction or valence bands and thereby modify the effective band structure of the film [17].

#### 3.1.2. Current–Voltage and Photoresponse Properties

**Figure 4:**
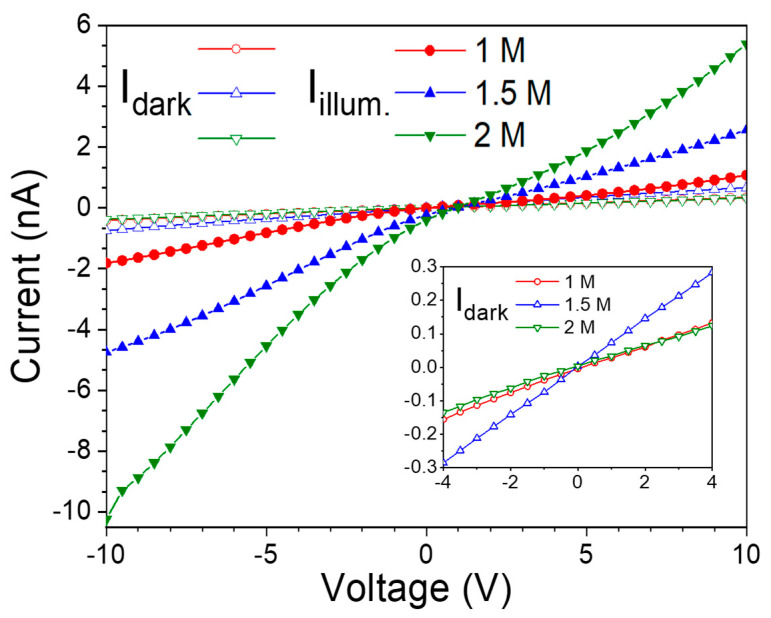
Dark currents and illuminated currents of CdS thin films prepared with different precursor concentrations.

To evaluate the electrical properties and photoresponse of CdS thin films prepared with varying precursor concentrations, interdigital aluminum electrodes were fabricated directly onto the surface of the nanostructured CdS films, forming meal–semiconductor–metal (MSM) devices. Current–voltage (I–V) measurements were performed under both dark and illuminated conditions. Illumination was provided by a monochromatic light source with a wavelength of 435 nm and an optical power of 1.15 µW. Figure 4 displays the I–V characteristics of the MSM devices based on CdS films, with the inset showing a magnified view of the dark current region. For all three concentrations, the dark I–V curves exhibit nearly linear behavior, indicating ohmic contact between the CdS film and aluminum electrodes. Among the tested samples, the device fabricated with the 2 M CdS film exhibited the lowest dark current, indicating reduced leakage and improved interface quality. In contrast, the photocurrent under illumination increased with precursor concentration, with the 2 M sample demonstrating the highest photocurrent.

These results indicate that the CdS thin film deposited using a 2 M precursor concentration exhibits superior photoresponse, which can be attributed to its improved crystallinity and the formation of a dense, pine-needle-like nanostructured surface. In addition, the presence of minor secondary phases, such as Cd(OH)_2_ and CdO, which formed during the CBD process under specific precursor concentration conditions, could influence the overall device performance. Cd(OH)_2_, an n-type semiconductor with high resistivity and a wide bandgap (~3.2–3.5 eV), may serve as a surface passivation layer when present in small amounts, thereby suppressing surface recombination and enhancing carrier lifetime [29]. CdO, on the other hand, with high conductivity and a bandgap of ~2.2–2.5 eV, can enhance current flow and contribute to additional absorption in the short-wavelength spectral region. Furthermore, its inclusion may potentially enhance charge separation and transport by modifying the interfacial band alignment within the heterojunction structure [30].

To further evaluate the photodetector performance, the spectral photoresponse was analyzed, and key figures of merit, including responsivity (*R*), specific detectivity (D*), and quantum efficiency (*η*) were calculated. The responsivity of the photodetector is defined as the ratio of the photocurrent (*I_illuminated_* − *I_dark_*) to the incident optical power (*P_in_*) over the effective device area. The responsivity is calculated according to Equation (5) [6]:

(5) $$R(\lambda )=\frac{I_{illuminated}(\lambda )-I_{dark}}{P_{in}}$$

where *I_illuminated_*(*λ*) is the current under illumination at wavelength *λ*, *I_dark_* is the dark current, and *P_in_* is the incident light power.

**Figure 5:**
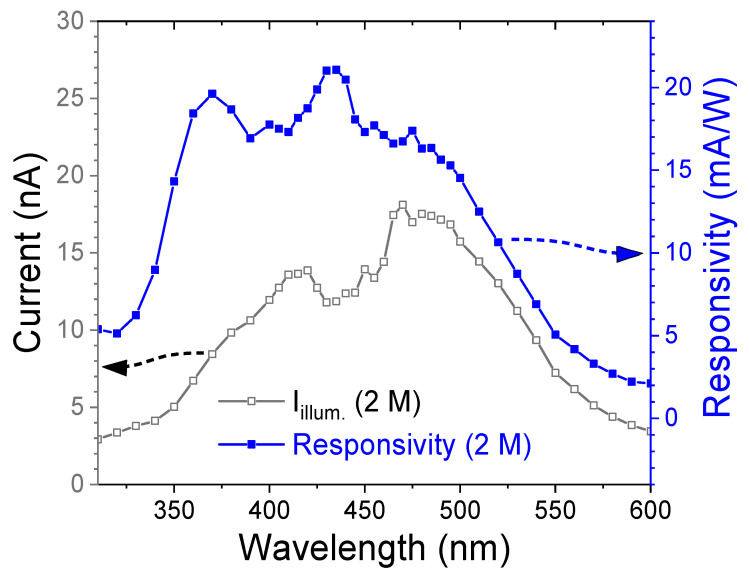
Illuminated current and responsivity of the nanostructured CdS thin film as a function of wavelength.

Figure 5 shows the illuminated current and spectral responsivity of the 2 M CdS thin film under a bias voltage of −10 V. The film exhibits a maximum responsivity of 21.1 mA/W in the blue spectral region (~435 nm) and a secondary high responsivity of 19.6 mA/W in the ultraviolet region (~370 nm). The responsivity decreases sharply in the green light region, reaching 2.10 mA/W at 600 nm.

Specific detectivity (D*) and quantum efficiency (*η*) are also critical parameters for evaluating photodetector performance. The specific detectivity represents the device’s ability to detect weak optical signals and was estimated under the widely adopted shot-noise-limited assumption, where the noise is dominated by shot noise from the dark current [31]. The quantum efficiency quantifies the effectiveness of converting incident photons into measurable electrical signals. These parameters are calculated using Equations (6) and (7) [6,32].

(6) $$D*=\frac{R\sqrt{S}}{\sqrt{2qI_{dark}}}$$

(7) $$\eta =\frac{R\cdot h\cdot c}{\lambda \cdot q}\times 100\%$$

where *R* is the responsivity, *S* is the effective illuminated area, *q* is the elementary charge (1.602 × 10^−19^ C), *I_dark_* is the dark current, *h* is Planck’s constant, *c* is the speed of light, and *λ* is the incident wavelength. The optimized photodetector based on the 2 M CdS thin film achieved a specific detectivity of 3.0 × 10^11^ Jones and a quantum efficiency of 6.01% at a wavelength of 435 nm, demonstrating its potential for short-wavelength light photodetection applications.

To evaluate and contextualize the performance of the nanostructured CdS thin film photodetector developed in this study, Table 2 summarizes a comparison of key device parameters with those reported for other CdS-based photodetectors. It is widely observed that the maximum photoresponse of most CdS-based photodetectors occurs within the short-wavelength spectral region, consistent with the intrinsic bandgap of CdS (~2.4 eV). Reported responsivity values for CdS photodetectors vary substantially, typically ranging from tens to thousands of mA/W. Notably, undoped CdS thin films fabricated via CBD generally exhibit lower responsivity compared to doped or 1D-structured CdS-based devices. Although the CdS films deposited with the optimized 2 M precursor concentration in this study demonstrated relatively high crystallinity in the hexagonal phase and better responsivity among the considered CBD samples, structural defects and secondary Cd compounds such as Cd(OH)_2_ and CdO were also detected within the film matrix. These inherent defects and multiphase components, when excessive, may adversely affect the generation and transport of photogenerated electron–hole pairs, thereby limiting the overall photocurrent.

**Table 2:** Comparison of the photodetector parameters obtained in this work with those reported in the literature for various CdS-based photodetectors.

Device Structure	Deposition Method	Wavelength, Voltage	R (mA/W)	D* (Jones)	Ref.
CdS	PLD ^1^	365 nm, 0 V	6210	-	[32]
CdS:Al (3%)	Spray	532 nm, 5 V	2130	5.2 × 10^11^	[33]
CdS:Na	Hydrothermal	390 nm, -	38	1.5 × 10^10^	[34]
FTO/CdS:Cl	CBD	365 nm, −2 V	570	3.7 × 10^8^	[35]
		530 nm, −2 V	1670	1.08 × 10^9^	
CdS:Mg (3%)	CBD	443 nm, 0 V	11	4.4 × 10^8^	[20]
Au/CdS/Au	Evaporation	420 nm, 10 V	380	2.6 × 10^13^	[36]
Al/CdS/Al	CBD	435 nm, −10 V	21.1	3.0 × 10^11^	This study

^1^ PLD, pulsed laser deposition.

To further enhance the responsivity, this work proposes the deposition of a p-type CuSCN thin film atop the nanostructured CdS layer to form a p–n heterojunction. This heterostructure is expected to effectively suppress dark current by reducing leakage pathways, while promoting more efficient separation and transport of photogenerated charge carriers.

### 3.2. P-N Heterojunction Photodetector

**Figure 6:**
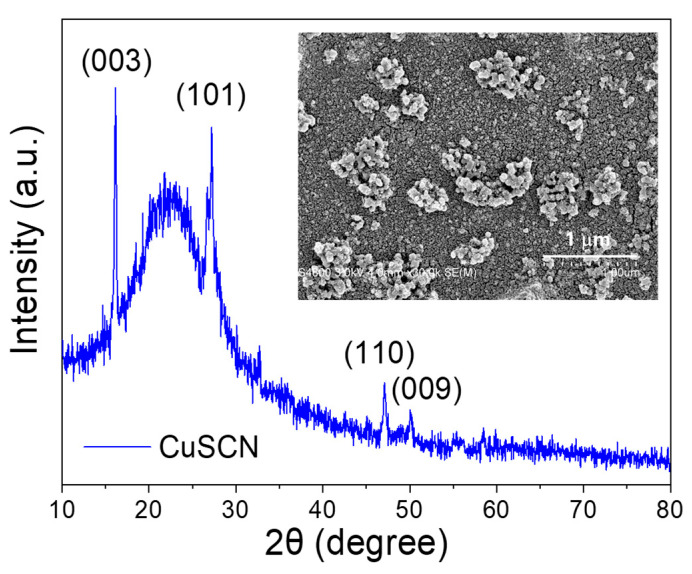
XRD pattern of the CuSCN thin film. The inset shows its plan-view FESEM image.

Figure 6 presents the XRD pattern of the CuSCN thin film. Distinct diffraction peaks are observed at 2θ = 16.12°, 27.24°, 47.23°, and 50.08°, corresponding to the (003), (101), (110), and (009) crystal planes, respectively. These peaks match the reference data from JCPDS No. 29-0581, confirming the rhombohedral crystal structure of CuSCN [37]. Notably, the peak at 2θ = 16.12° exhibits the highest intensity, indicating a preferred orientation along the (003) plane in the deposited film. The inset of Figure 6 shows the surface morphology of the CuSCN film, as characterized by FESEM. The film—formed through 20 sequential spin-coating cycles—displays a highly compact and dense surface without visible pinholes. However, nanoscale particles are observed to aggregate into clusters, distributed non-uniformly across the surface. This morphological feature is consistent with the findings of Neeraj et al. [18], who reported uneven nanostructure distributions in CuSCN films fabricated via solution-based deposition. These observations suggest that repeated spin-coating cycles may enhance surface agglomeration, leading to cluster formation from nanoparticle accumulation.

**Figure 7:**
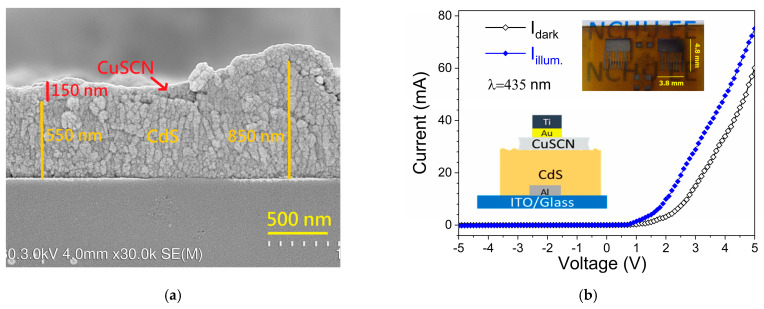
(**a**) Cross-sectional SEM micrograph, (**b**) I–V curves of the p-CuSCN/n-CdS heterojunction device. The inset shows the schematic diagram and the photograph of the device.

The p-CuSCN/n-CdS heterojunction photodetector was constructed using an Al/ITO bilayer as the bottom electrode and a Ti/Au bilayer as the top contact. Figure 7a shows a cross-sectional SEM image of the CdS/CuSCN heterostructure. The nanostructured CdS film exhibits a thickness ranging from approximately 550 to 850 nm, while the spin-coated CuSCN layer is uniformly deposited with a thickness of around 150 nm. Figure 7b displays the I–V characteristics of the device under both dark and illuminated conditions. The inset includes the schematic diagram and the photograph of the developed semi-transparent p-n heterojunction device. The dark I–V curve shows a clear diode-like rectifying behavior, confirming the successful formation of the p–n junction. Notably, the rectification ratio reaches approximately 750 at ±5 V, indicating strong asymmetric current transport across the junction. Compared to the MSM-structured CdS device (Figure 4), which exhibits a dark current of 1.88 nA at +5 V, the p–n device shows a significantly higher current of 60.1 mA under the same bias. This substantial enhancement is attributed to improved charge injection and carrier transport facilitated by the CuSCN/CdS heterostructure, highlighting the benefits of heterojunction engineering for performance optimization.

The ideality factor (n) of the diode was extracted from the forward I–V characteristics based on the Shockley diode Equation (8):

(8) $$I=I_{0}\left[exp\left(\frac{qV}{nkT}\right)-1\right]$$

where *I*_0_ is the reverse saturation current, *q* is the elementary charge, *k* is the Boltzmann constant (1.381 × 10^−23^ J/K), and *T* is the absolute temperature in Kelvin. By performing a linear fit of ln(I) versus V in the exponential region, the ideality factor was calculated from the slope (*q*/n*kT*). The resulting ideality factor is 1.39. An ideality factor close to unity suggests that carrier transport is primarily dominated by diffusion. The slight deviation from the ideal value (n = 1) may be attributed to the presence of interface states or recombination of minority carriers in the depletion region—phenomena commonly observed in heterojunction devices [38,39].

The turn-on voltage of the device was determined by extrapolating the linear portion of the forward I–V curve to the *x*-axis, yielding a value of approximately 1.94 V. This relatively high turn-on voltage is likely due to the significant band offset between CuSCN and CdS, which contributes to a large built-in potential across the heterojunction. Similar behavior has been observed in other systems, where intentional band alignment engineering improves rectification performance at the cost of a higher conduction threshold [40]. These findings confirm that the CdS/CuSCN heterostructure forms a robust rectifying junction with a high current density and a strong built-in field, which is beneficial for photogenerated carrier separation and improves the photoresponse.

**Figure 8:**
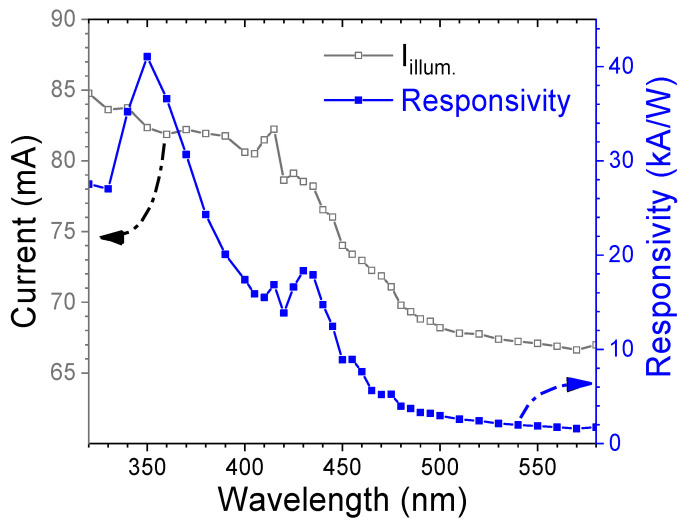
Illuminated current and responsivity of the p-CuSCN/n-CdS heterojunction diode as a function of wavelength.

To evaluate the spectral photoresponse, the fabricated p-CuSCN/n-CdS heterojunction photodetector was illuminated under UV–visible light across a broad wavelength range. The illuminated current and calculated responsivity are plotted in Figure 8. The device exhibits significantly enhanced photocurrent in the short-wavelength region, with a sharp decline in response beyond 440 nm. It is worth noting that the responsivity, calculated using Equation (5), exceeds 10^4^ A/W in the short-wavelength region (λ < 440 nm) and reaches a peak of 4.11 × 10^4^ A/W at 350 nm. The enhanced UV response is primarily attributed to the near-band-edge absorption of CuSCN, a wide-bandgap semiconductor with a bandgap in the range of 3.6–3.9 eV [41], in combination with the strong UV absorption capability of nanostructured CdS layer. Sancan Han et al. also reported that the p-CuSCN film displayed better UV photoresponse (at ~340 nm) than blue light response [42]. Meanwhile, the blue-light responsivity is primarily associated with defect-mediated absorption within the nanostructured CdS film or at the CdS/CuSCN interface.

Compared with the CdS-based MSM photodetector (Figure 5), the responsivity of the heterojunction device in the short-wavelength region is enhanced by more than six orders of magnitude. This dramatic improvement stems from the synergistic optical absorption of both CuSCN and CdS, and the efficient generation of electron–hole pairs within the extended depletion region of the p-n junction. The strong built-in electric field across the junction facilitates rapid carrier separation, thereby minimizing recombination losses. Additionally, CuSCN serves as an effective hole transport layer (HTL), enhancing hole extraction and transport while further suppressing carrier recombination. The heterojunction device exhibits a maximum specific detectivity (D*) of 7.92 × 10^13^ Jones, demonstrating its high sensitivity to weak light. These performance metrics confirm that the p-CuSCN/n-CdS heterojunction is a promising candidate for high-responsivity photodetection in the short-wavelength spectral ranges.

Compared with previously reported high-responsivity (R > 10 A/W) CdS heterostructure-based photodetectors (summarized in Table 3), which are almost all based on 1D nanostructures prepared by CVD or PVD, the photodetector proposed in this study adopts a cost-effective solution-processed fabrication route while providing excellent performance. This highlights the great potential of the CdS/CuSCN heterojunction architecture in next-generation, low-cost, high-performance optoelectronic applications.

**Table 3:** Comparison of the main parameters of this study with previously reported high-response CdS heterostructure-based photodetectors.

Device Structure	Deposition Method	Wavelength, Voltage	R (mA/W)	D* (Jones)	Ref.
CdS NR/Cs_2_AgBiBr_6_	PVD	440 nm, 3 V	6.66 × 10^3^	2.10 × 10^14^	[43]
CdS*_x_*Se_1−_*_x_*NR/PbI_2_ NS	PVD	600 nm, 2.5 V	567	1.96 × 10^15^	[44]
CdS NR/CdS*_x_*Se_1−_*_x_* NR	PVD	510 nm, 1 V	1.5 × 10^5^	5.2 × 10^11^	[45]
CdS-CdSTe-CdTe NBs	CVD	405 nm, 5 V	1.52 × 10^3^	1.5 × 10^10^	[46]
CdS/CdS*_x_*Se_1−_*_x_* NWs	CVD	500 nm, 1 V	118	-	[47]
CdS NWs/Te NFs	CVD	450 nm, 1 V	126	1.03 × 10^10^	[48]
CdS NWs/GeS CS	CVD	405 nm, 1 V	76.8	-	[49]
CdS/ZnO NRs	CBD	350 nm, 5 V	12.9	-	[50]
CdS/CuSCN	CBD	350 nm, 5 V	4.11 × 10^4^	7.92 × 10^13^	This study

NR, nanoribbon; NS, nanosheet; NRs, nanorods; NWs, nanowires; NFs, nanoflakes; CS, core–shell.

## 4. Conclusions

In this work, the influence of precursor concentration on the structural, optical, electrical, and photoresponse characteristics of chemically bath-deposited (CBD) nanostructured CdS thin films was systematically investigated. The MSM photodetector fabricated using a 2 M precursor solution exhibited a responsivity of 21.1 mA/W at 435 nm, which is attributed to enlarged grain size, a densely packed pine needle-like surface morphology, and superior absorption. Building upon these optimized nanostructured films, a high-performance p–n heterojunction photodetector was successfully fabricated by integrating solution-processed n-CdS and p-CuSCN layers. The resulting device demonstrated excellent diode behavior with a rectification ratio of ~750 and an ideality factor of 1.39. Spectral analysis revealed a peak responsivity of 4.11 × 10^4^ A/W under a 5 V bias, surpassing that of the CdS-based MSM device by over six orders of magnitude. Moreover, the device achieved a high specific detectivity of 7.92 × 10^13^ Jones in the UV region. These findings highlight the great potential of solution-processed CdS/CuSCN heterostructures as low-cost, high-sensitivity photodetectors, suitable for next-generation applications in environmental sensing, portable UV monitoring, and optoelectronic systems.

## Data Availability

The data that support the findings of this study are available within the article.
